# YES! YOU CAN DO DAILY DIARY RESEARCH WITH DEMENTIA CAREGIVERS!

**DOI:** 10.1093/geroni/igad104.1973

**Published:** 2023-12-21

**Authors:** Vicki Winstead, Carolyn Pickering, Vicki Winstead

## Abstract

Successful recruitment and retention of dementia caregivers for intensive research protocols such as daily diary research is thought to be hindered by factors that include having limited time and the stress in dementia caregiving. Knowing why caregivers participate and what keeps them motivated to comply in daily diary research is crucial in understanding the experiences of dementia caregiving for the purpose of developing and ensuring successful interventions. The purpose of this symposium is to present data from three online micro-longitudinal daily diary studies on caregiving and dementia that inform successful practices in recruitment, compliance and reducing missing data in dementia caregivers. Each of the studies required daily participation ranging from 1) four daily diaries each day over 8 consecutive days, 2) 21 consecutive days of diaries once per day, and 3) three different data collection points of 21 consecutive days over one year. The symposium includes three papers that address the following areas: 1) Identifying factors that motivate dementia caregivers to participate in research for the purpose of developing relevant and meaningful recruitment materials. 2) Describing and predicting “missingness” within diary research, and 3) Effective strategies that are intentional in promoting high levels of compliance in completing diaries. Discussion will include ways to enhance recruitment materials that are congruent with caregivers’ motivations to participate, how strategies were used in each of the three studies for compliance in completion of daily surveys and retention and provide evidence that caregivers do take the time to participate in intensive research protocols.

